# Repercussions of the COVID-19 pandemic on the well-being and training of medical clerks: a pan-Canadian survey

**DOI:** 10.1186/s12909-020-02293-0

**Published:** 2020-10-27

**Authors:** Myriam Abbas, Malek Dhane, Michèle Beniey, Léamarie Meloche-Dumas, Mohamed Eissa, Natasha Guérard-Poirier, Myriam El-Raheb, Florence Lebel-Guay, Adam Dubrowski, Erica Patocskai

**Affiliations:** 1 Simulation and Medical Education Research Group, Université de Montréal, Montréal, QC Canada; 2 Faculté de médecine, Université de Montréal, 2900 Blvd Edouard Montpetit, Montréal, Quebec H3T 1J4 Canada; 3 Faculty of Medicine, Dalhousie University, 6299 South Street, Halifax, Nova Scotia B3H 4R2 Canada; 4 Ontario Tech University, 200 Simcoe Nord street, Oshawa, Ontario L1G 0C5 Canada; 5 Department of Surgical Oncology, Centre Hospitalier de l’Université de Montréal (CHUM), 1051 rue Sanguinet, Montréal, Quebec H2X 0C1 Canada

**Keywords:** COVID-19, Medical education, Student well-being, Clerkship, Medical training

## Abstract

**Background:**

The COVID-19 pandemic has been an unprecedented and potentially stressful event that inserted itself into the 2019–2020 Canadian medical curriculum. However, its impact on stress and subsequent professional pathways is not well understood. This study aims to assess the impact of the COVID-19 pandemic on the mental well-being, training, and career choices of Canadian medical clerks within the first three months of the pandemic. It also aims to assess their use of university support systems and their appreciation of potential solutions to common academic stressors.

**Methods:**

An electronic survey composed of four sections: demographics, stressors experienced during the pandemic, World Health Organization (WHO) well-being index, and stress management and resources was distributed to Canadian clerks.

**Results:**

Clerks from 10 of the 17 Canadian medical faculties participated in this study (*n* = 627). Forty-five percent of clerks reported higher levels of stress than usual; 22% reconsidered their residency choice; and 19% reconsidered medicine as a career. The factors that were most stressful among clerks were: the means of return to rotations; decreased opportunities to be productive in view of residency match; and taking the national licensing exam after the beginning of residency. The mean WHO well-being index was 14.8/25 ± 4.5, indicating a poor level of well-being among a considerable proportion of students. Clerks who reconsidered their residency choice or medicine as a career had lower mean WHO well-being indices. Most clerks agreed with the following suggested solutions: training sessions on the clinical management of COVID-19 cases; being allowed to submit fewer reference letters when applying to residency; and having protected time to study for their licensing exam during residency. Overall, clerks were less concerned with being infected during their rotations than with the impact of the pandemic on their future career and residency match.

**Conclusion:**

The COVID-19 pandemic had a considerable impact on the medical curriculum and well-being of clerks. A number of student-identified solutions were proposed to reduce stress. The implementation of these solutions throughout the Canadian medical training system should be considered.

## Background

The well-being of university students has been a subject of particular concern in recent times. A national study conducted in Canada found that 30% of undergraduate university students reported elevated psychological distress, which was significantly higher than the general population [1]. Medical trainees are no exception to these statistics as they report a high prevalence of anxiety and stress [2, 3].

Evidence suggests that these trends among medical students tend to worsen during their medical curriculum, with a significant increase of perceived stress among third-year medical students when compared to their first-year of medical school [4]. High levels of stress and anxiety were correlated with depression and burnout [2, 5]. One study found that 43% of third year and 31% of fourth year medical students experienced moderate or high degree of burnout [6]. The deleterious impact of this psychological distress on clerks’ lives has been well documented, such as a detrimental effect on cognitive functioning and academic performance, as well as intention of dropping out [7–9].

An unprecedented and potentially stressful event that inserted itself into the 2019–2020 medical curriculum is the COVID-19 pandemic. The impact of this pandemic on the wellbeing of health-care workers has already been well documented [10–12]. Yet, its impact on stress and subsequent professional pathways for medical trainees is still not well understood. During the pandemic, although the academic curriculum of preclinical students continued online, the situation was different for most medical clerks. In many countries, senior clerks had to graduate prematurely, [13] while, in other parts of the world, clerks were pulled from their respective rotations. In Canada, most junior and senior clerks saw their medical rotations be suspended.

The Canadian medical curriculum follows a traditional format where students in third-year transition to clerkship in a teaching hospital. The impact of this pandemic may be particularly pronounced for students during clerkship who are preparing for their future careers. In fact, in Canada, it is during clerkship that third-year medical students, also known as junior clerks, choose electives and obtain reference letters from attendings in order to apply to residency. On the other hand, fourth-year medical students, known as senior clerks, are tasked with completing the national licensing exam. However, due to the pandemic, the specific requirements and dates of the Canadian Resident Matching Service (CaRMS) and the Medical Council of Canada Qualifying Examination (MCCQE) are being reconsidered.

Given their known predisposition to decreased mental well-being, [2, 14, 15] this study aims to assess the impact of the COVID-19 pandemic on the mental well-being, training, and career choices of Canadian medical clerks within the first three months of the pandemic. It also aims to assess the use of mental well-being support systems, as well as their appreciation of potential solutions to common academic stressors.

## Methods

### Study design and participants

An electronic survey composed of four sections: demographics, stressors and their impact on residency and career choice, World Health Organization (WHO) well-being index and stress management and resources was distributed to clerks (third- and fourth-year medical students) in all Canadian medical schools, with 10 of the 17 faculties participating and forwarding the survey to their clerks. The survey was developed and delivered in French and English. The study protocol, consent form and recruitment documents were approved by the institutional review board of the Université de Montréal (CERSES-20-060-D).

Eligible participants were clerks attending one of the 17 Canadian medical faculties, and whose rotations were suspended due to the COVID-19 pandemic. Clerks whose clerkship had been suspended for any other reason were excluded.

### Survey development

The survey (Additional file 1) contained four sections: (1) Demographics, (2) Stressors (3) WHO well-being index, and (4) Stress management and resources. These four sections of the survey were developed by either adapting existing sources [16] or by conducting short focus group sessions with a small sample of junior and senior clerks from a single Canadian medical school.

#### Demographics

The first section of the survey consisted of questions on demographics; age, gender, level of education, university, implications and occupations, COVID-19 infection status and preferred residency. Senior clerks were asked about the specialty to which they had already matched.

#### Stressors

Stressors included in the survey were developed with a focus group of Canadian medical clerks. This focus group was composed of four third year medical students and two fourth year medical students whose clinical rotations were suspended due to the COVID-19 pandemic. A meeting was held in order to select the main stressors by reflecting on their own experience as clerks and the experience of their classmates.

This section included an initial question allowing an assessment of the respondent’s stress level in the context of the COVID-19 pandemic. The three subsequent questions assessed the impact of the pandemic on their choice of residency and their decision to pursue a medical career. If the respondent reconsidered his residency choice, an additional question was asked to see the type of residency change they considered. This section also contained a question allowing the respondent to rate, on a Likert scale, the level of stress associated with potential stressors they may have experienced. Most of these stressors were directly linked to future educational and professional pathways.

#### WHO well-being index

The third section of the survey assessed the respondent’s state of well-being by using the WHO well-being index [16]. This measurement tool consists of five statements that respondents apply to their own lives*.* It has high clinical validity and is among the most widely used questionnaires assessing subjective psychological well-being [17]. It has been translated into over 30 languages, including French [17]. The maximum score of this index is 25 and a score of 13 or lower indicates a poor state of well-being [18].

#### Stress management and resources

The fourth section of the survey contained five questions evaluating whether the respondent used university psychosocial resources during the COVID-19 pandemic, as well as the perceived usefulness of the resource. This section also included a question exploring the level of agreement regarding certain academic solutions. The level of agreement with each solution was assessed using a 5-point Likert scale ranging from *“Strongly agree”* to *“Strongly disagree”.* A final open-ended question allowed respondents to offer other potential solutions.

In order to ensure validity of the survey, its contents were reviewed by a group of experts composed of members of the medical stimulation and education research group at the Université of Montréal and the Ontario Tech University, consisting of physicians, researchers in medical education, residents, and medical students.

### Recruitment and distribution of the survey

The survey was developed and distributed using the Qualtrics XM platform [11]. The request to participate was sent to the deans and curriculum directors of all 17 Canadian medical faculties. They were asked to review the summary of the study and the survey and decide if the faculty would participate. Because the aim of this study was to capture the well-being, stressors, and management within the acute stage of the COVID-19 pandemic, we requested a response within a month. For the faculties that agreed to participate, the clerkship directors were asked to send the survey to all eligible clerks by distributing an email containing a digital link. For faculties that chose not to participate, the clerkship directors were asked for a short explanation and reasoning for not participating in the study.

Participation in this study was voluntary and consent was obtained with a consent form at the beginning of the survey. The survey was anonymous and took 10 to 15 min to complete.

### Data collection and statistical analysis

The study was conducted between April 9th, 2020 and June 3rd, 2020.

Data collected for each of the four sections of the survey were analyzed separately. The data was first cleaned and analyzed in Microsoft Excel. Except for participants’ characteristics, missing data from participants who elected not to answer certain questions were excluded from all calculations.

#### Demographics

Categorical variables (age, level of study, home university, preferred residency match, COVID status, and family COVID status) were described as proportions of the study population.

#### Stressors

Descriptive statistics were performed. Proportions were used to assess the number of clerks who reconsidered medicine as a career and their residency choice, and to assess levels of stress during the pandemic.

#### WHO well-being index

First, we assessed the frequency distribution of the index and performed descriptive statistics for all participants with the mean and standard deviation. Second, we compared the mean WHO well-being index between two groups of participants: participants who reconsidered medicine as a career and participants who did not. This data was obtained from part 2 of the survey (Stressors). We used unpaired t-tests to compare these two means. For each analysis, confidence intervals were computed for a 95%-degree confidence. *P*-values less than 0.05 were interpreted as indicating a statistical difference*.* All inferential statistics were performed in GraphPad Prism [19].

#### Stress management and resources

Descriptive statistics were performed using proportions. For the final open-ended question, responses were grouped by theme. The themes that came up more than ten times by different students were considered valid solutions.

## Results

Of the 17 Canadian medical faculties, 10 accepted to participate and were able to send the survey to their students within an acceptable timeframe. Of the seven faculties that chose not to participate, one did not reply, two chose not to distribute the survey without providing a reason, and four chose not participate because their institutional research ethics board reviewing process could not meet the deadline of the study. There were 677 responses, of which 48 (8.0%) were excluded because their clerkship rotations had not been suspended due to COVID-19, and two did not provide informed consent. Therefore, a total of 627 participants were included in the final survey.

### Demographics

Table 1 shows the major characteristics of the participants. The majority of the included participants were female (*n* = 439, 70.7%). During the confinement period, most clerks resided at home with their parents (*n* = 252, 40.9%) or with a partner (*n* = 186, 30.2%). A minority of respondents were taking care of a dependent, including a child and/or an elderly person (*n* = 34, 5.5%).

**Table 1:** Characteristics of the participants

Characteristics	Number	Percent
**Age (years)**		
20–25	388	(61.9)
25–30	199	(31.7)
30–35	27	(4.3)
More than 35	9	(1.4)
Unknown	4	(0.6)
**Level of study**		
Junior clerks	469	(74.8)
Senior clerks	151	(24.1)
Unknown	7	(1.1)
**Home University**		
Dalhousie University	38	(6.1)
Université Laval	22	(3.5)
University of Manitoba	39	(6.2)
Memorial University of Newfoundland	16	(2.6)
Université de Montréal	188	(30.0)
Northern Ontario School of Medicine	9	(1.4)
University of Ottawa	46	(7.3)
Queen’s University	18	(2.9)
Université de Sherbrooke	118	(18.8)
University of Toronto	123	(19.6)
Unspecified	10	(1.6)
**Preferred residency match**		
Family medicine	188	(30.0)
Medical specialty	422	(67.3)
Unknown	17	(2.7)
**COVID status**		
Positive with mild to moderate symptoms	3	(0.5)
Positive with severe symptoms^a^	0	(0.0)
Negative	613	(97.8)
Unknown	11	(1.8)
**Family COVID status**		
Positive with mild to moderate symptoms	13	(2.1)
Positive with severe symptoms^a^	6	(1.0)
Negative	593	(94.6)
Unknown	15	(2.4)

Abbreviations:

^a^Severe symptoms defined as requiring hospitalisation or ICU stay

The vast majority of students reported partaking in hobbies and/or self-care activities during the pandemic (*n* = 590, 96.8%) and most were involved in volunteering (*n* = 319, 52.6%). Only a minority of students participated in remunerated work outside of clerkship (*n* = 122, 20.3%). Many students had already had online medical classes at some point during clerkship suspension (*n* = 446, 73.5%).

### Stressors

**Fig. 1:**
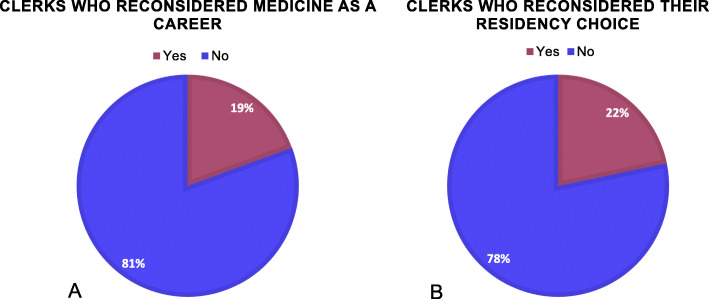
Participants who reconsidered **a**) medicine and **b**) their residency choice during the pandemic

**Fig. 2:**
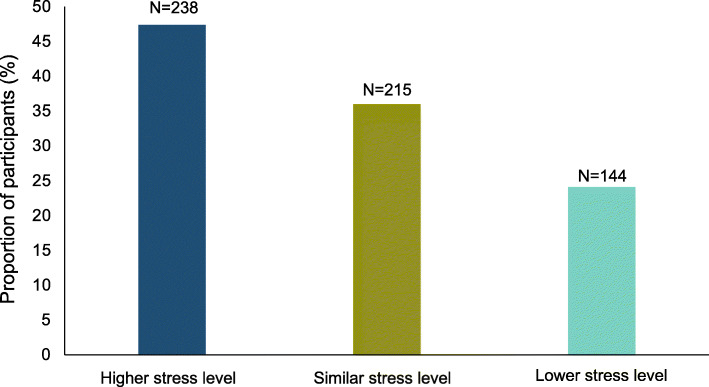
Stress levels among the participants during the pandemic

**Fig. 3:**
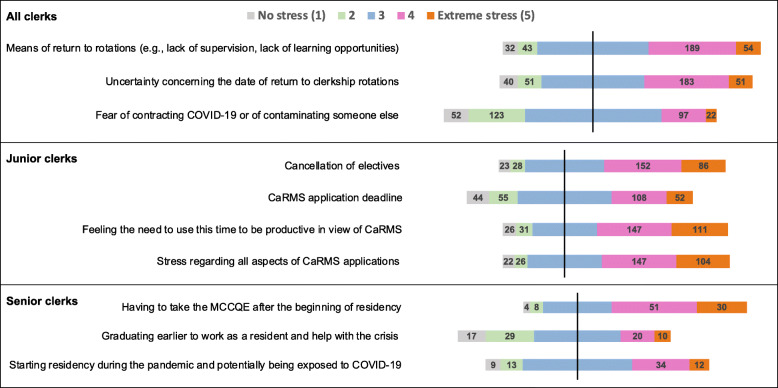
Likert scales of identified stressors. Abbreviations: CaRMS: Canadian Resident Matching Service. COVID: Coronavirus disease. MCCQE: Medical Council of Canada Qualifying Examination

Figure 1 reports that 116 clerks (19%) reconsidered medicine as a career, and 130 clerks (22%) reconsidered their residency choice. Figure 2 shows the clerks’ level of stress during the pandemic with 238 clerks (40%) reporting higher levels of stress than usual. Figure 3 describes participants’ self-reported level of stress for various stress factors. The factor that was the most stressful for all clerks was the means of return to rotations (e.g., lack of supervision, lack of learning opportunities). The most stressful factors for junior clerks were feeling the need to stay productive in view of CaRMS, and stress regarding all aspects of CaRMS application. For senior clerks, having to take the MCCQE after the beginning of residency was the most stressful factor.

### WHO well-being index

The overall mean WHO well-being index of the included participants was 14.8 ± 4.5, indicating a poor level of well-being among a considerable proportion of students. Figure 1 shows the proportion of participants who reconsidered their medical career (*n* = 116) and those who reconsidered their choice of residency (*n* = 130) during the pandemic. Participants who reconsidered medicine as a career had a mean WHO well-being index of 13.5 ± 4.6. This value was significantly different when compared to participants who did not reconsider medicine (15.2 ± 4.4; t (590) = 3.67, *p* = 0.0003). The mean WHO well-being index of participants who reconsidered their choice of residency was 13.5 ± 4.2 with a statistically significant difference when compared to participants who did not reconsider their choice of residency (15.2 ± 4.5, t (590) = 4.04, *p* < 0.0001).

### Stress management and resources

**Fig. 4:**
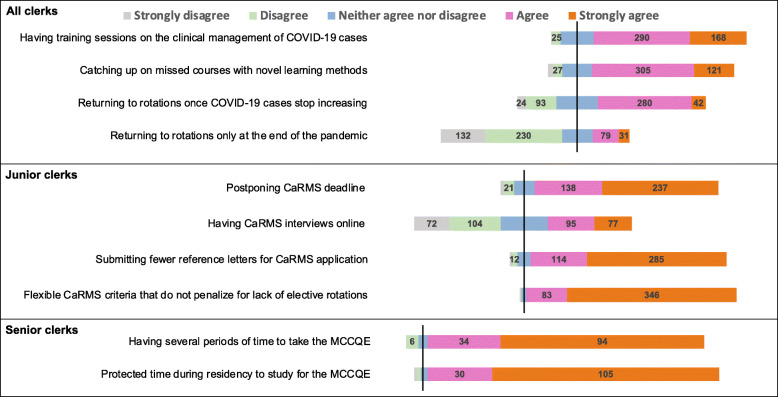
Likert scales of potential solutions. Abbreviations:CaRMS: Canadian Resident Matching Service. COVID: Coronavirus disease. MCCQE: Medical Council of Canada Qualifying Examination

Of the included participants, 69 (11.7%) reported using various university mental health support resources to assist them during the pandemic. Of these students, 31 (44.9%) did not find the resource they used helpful. Figure 4 depicts the possible solutions to the stressors for junior and/or senior clerks. Junior and senior clerks were highly in favor of having training sessions on the clinical management of COVID-19 cases. Among the solutions that were proposed for junior clerks, solutions that were highly appreciated were flexible CaRMS criteria that do not penalize for lack of elective rotations and submitting fewer reference letters for CaRMS application. Senior clerks were highly in favor of having protected time during residency to study for the MCCQE and having several periods of time to take the MCCQE.

Solutions that were frequently cited in the final open-ended question of the survey were: having more transparency concerning decisions taken regarding the medical curriculum, canceling end of training examinations, ensuring an adequate amount of elective weeks following the pandemic and CaRMS interviews excluding questions about the applicants’ activities during the clerkship suspension.

## Discussion

With the recent COVID-19 pandemic sweeping across Canada, medical clerks were removed from clinical rotations and had their clerkship suspended, missing-out on valuable clinical experience [20]. Our study aimed to assess the impact of the COVID-19 pandemic on the mental well-being, training, and career choices of Canadian medical clerks. It also aims to assess the use of mental well-being support systems, as well as their appreciation of potential solutions to common academic stressors.

The need to adapt to new environments is one of the challenging elements of medical students’ educational journey [7, 21]. The relative lack of tolerance for uncertainty and ambiguity has been found to be an important predictor of psychological distress among medical students [22]. Our study showed that approximately 45% of surveyed students had higher stress levels than usual during the pandemic (Fig. 2). This may be due to the challenge of facing such a unique event.

Historically, there has been very few dropouts from Canadian medical schools. According to the Association of Faculties of Medicine of Canada, only 0.5% of students had dropped out of a medical school in 2017 [23]. As shown in Fig. 1, our study found that 19% of surveyors reconsidered medicine as a career during this pandemic, with 22% reconsidering their choice of residency. This could be due to limited clinical exposure, or perhaps the fear of not being competitive for selective residencies [24]. However, this change in perspective may also be due to their mental state during the pandemic. In fact, the results of the third part of our survey showed that a considerable amount of respondents had a WHO well-being index below 13, which suggests poor well-being and would require psychological support [17]. Furthermore, clerks who reconsidered medicine as a career or reconsidered their residency choice had significantly lower mean WHO well-being indices. Yet, our results showed that only a minority of students used a university support system, and of those who used a resource, half regarded it as unhelpful. This begs the question on whether the resources currently available to medical clerks are both accessible and well-tailored to their needs.

Our study also identified several factors that could explain this increase in stress levels during the pandemic (Fig. 3). Both junior and senior clerks were found to have increased stress with respect to means of clerkship resumption. As shown in Fig. 4, the majority of clerks were not in favour of the resumption of rotations only at the end of the pandemic. Instead, returning when cases cease to increase was deemed more favorable to them. Interestingly, clerks were less anxious about contracting COVID-19 during their rotations despite the inherent risk, which challenges the pertinence of suspending clerkship altogether. Indeed, as evidenced by Fig. 3, clerks were more concerned with their eventual return to rotations and the impact of the pandemic on their education and future careers than with contracting the disease or contaminating others. According to the demographics part of our survey, clerks were also more willing to stay active during clerkship suspension as more than half decided to volunteer during the crisis. It could therefore be argued that suspending rotations may not have been in the clerk’s best interest. That being said, faculties made such a decision with more than just the interest of their students’ in mind, as reducing the amount of vectors in clinical settings was pivotal to flattening the curve [25].

Among junior clerks, the need to stay productive during the pandemic in view of CaRMS was a major source of anxiety (Fig. 3). Literature has shown that medical students utilize clerkship to make career decisions [26]. It also provides students with a chance to undertake projects and obtain reference letters from senior staff members to help support their residency applications. With clerkship suspension, clerks were impeded from participating in patient care and working with attendings or residents [27]. Junior clerks were also in favour of flexibility so as not to be penalized for a lack of electives in their specialty of choice (Fig. 4). This is particularly important as many faculties have decided to prioritize core rotations, and the possibility of doing electives outside one’s home university has been suspended for an undetermined amount of time across the country [20].

Regarding senior clerks, a major stress factor was writing the MCCQE part I during residency (Fig. 3). Traditionally, senior clerks were able to write the exam prior to starting their residency. However, due to social distancing rules, many test centers were shut down causing postponement of the exam [28]. At the time of the survey, the majority of clerks were in favour of having multiple time periods to take the MCCQE Part I examination and having protected time during residency to study for it (Fig. 4). Surprisingly, as shown in Fig. 3, graduating prematurely to actively help during the crisis was not a major source of stress. This is in keeping with the notion that most students were not afraid of contracting the disease or contaminating others.

As part of the solutions that were explored in our study, the majority of clerks were highly in favor of novel learning methods (Fig. 4). For many years, medical faculties have been aiming to incorporate technology into their teaching methods, and with the pandemic many were able to quickly transition their teaching sessions into online formats [27]. This can provide a solid foundation to permanently incorporate remote learning in medicine.

A limitation in our study is that medical schools in Alberta, British Colombia and Saskatchewan are not represented. Also, participating in the survey was optional and may have introduced a selection bias. Furthermore, some elements of the survey were designed to be as specific as possible to the reality of Canadian medical clerks. As such, not all means of measuring outcomes utilized validated questionnaires and may have introduced response bias. Overall, the results of this survey should be viewed as a qualitative and descriptive assessment of the general impact of the pandemic on Canadian clerks rather than an analytical assessment.

## Conclusion

The COVID-19 pandemic has forced medical students to adapt to a unique situation in order to achieve their educational goals. The pandemic resulted in considerable stress among clerks which medical faculties should address. Various solutions regarding the medical curriculum were viewed more favourably than others. This study can serve as a reference for faculty leaders when taking important decisions on behalf of their students.

## Supplementary information

Additional file 1. (DOCX 24 kb)

## Data Availability

The datasets used and/or analysed during the current study are available from the corresponding author on reasonable request.

## Abbreviations

COVID-19: Coronavirus disease 2019
WHO: World Health Organization
CaRMS: Canadian Resident Matching Service
MCCQE: Medical Council of Canada Qualifying Examination
