# Supplementary material for: Repercussions of the COVID-19 pandemic on the well-being and training of medical clerks: a pan-Canadian survey
Source: BMC Med Educ. 2020 Oct 27;20:385. doi: 10.1186/s12909-020-02293-0 (PMC7590563; doi:10.1186/s12909-020-02293-0)
Supplement: Supplementary file 1 — (DOCX 24 kb) [file 12909_2020_2293_MOESM1_ESM.docx]

**Appendix 1**

**Repercussions of the COVID-19 Pandemic on the Well-Being and Training of Medical Clerks: A Pan-Canadian Survey**

1.Have your clerkship rotations been suspended because of COVID-19?

Yes

No

**1-Demographics**

2.Age :

Less than 20 years old

20-25 years old

25-30 years old

30-35 years old

More than 35 years old

3. Sex:

Male

Female

Prefer not to say

4. Current level of study

Junior clerk

Senior clerk

5. Home University

1= University of Alberta

2= University of British Columbia

3= University of Calgary

4= Dalhousie University

5= Université Laval

6= University of Manitoba

7= McMaster University

8= McGill University

9= Memorial University of Newfoundland

10= Université de Montréal

11= Northern Ontario School of Medicine

12= University of Ottawa

13= Queen’s University

14= Université of Saskatchewan

15= Université de Sherbrooke

16= University of Toronto

17= Western University

6. Who are you currently residing with?

A) Alone

B) With parents

C) With partner

D) With partner and children

E) With children

F) With a room-mate

G) Other (specify): __________________

7. Are you taking care of any dependants?

A) Yes (e.g. child, and/or an elderly person)

B) No

8. At this point in time, have you contracted COVID-19 (confirmed with microbiologic testing)?

A) Yes, I had mild to moderate symptoms.

B) Yes, I had severe symptoms (i.e., requiring hospitalization or a stay in the intensive care unit).

C) No, I have not.

9. Has a member of your family contracted COVID-19? (Multiple answers allowed)

A) Yes, a member of my family contracted COVID-19 and developed mild to moderate symptoms.

B) Yes, a member of my family contracted COVID-19 and developed severe symptoms (i.e., requiring hospitalization, a stay in the intensive care unit or resulted in his/her death).

C) No, no member of my family has contracted COVID-19.

10. Since rotations have been suspended, have you participated in any of the following activities?

- Online medical classes: Yes or No

- Volunteering (e.g., call center, supporting staffs and residents, Public health office): Yes or No

- Remunerated work outside of clerkship: Yes or No

- Hobbies and self care activities: Yes or No

11. In which residency program would you like to match into or would you have liked to match into?

A) Anatomical pathology

B) Anesthesiology

C) Cardiac surgery

D) Dermatology

E) Diagnostic Radiology

F) Emergency medicine

G) Family medicine

H) Genetics

I) General surgery

J) Internal medicine

K) Neurosurgery

L) Neurology

M) Nuclear medicine

N) Obstetrics and gynecology

O) Ophthalmology

P) Orthopedic surgery

Q) Otolaryngology – Head and neck surgery

R) Pediatrics

S) Physical medicine and rehabilitation

T) Plastic surgery

U) Psychiatry

V) Public health and preventive medicine

W) Radiation Oncology

X) Urology

Y) Vascular Surgery

12. If you are a senior clerk, what residency program did you match into?

A) Anatomical pathology

B) Anesthesiology

C) Cardiac surgery

D) Dermatology

E) Diagnostic Radiology

F) Emergency medicine

G) Family medicine

H) Genetics

I) General surgery

J) Internal medicine

K) Neurosurgery

L) Neurology

M) Nuclear medicine

N) Obstetrics and gynecology

O) Ophthalmology

P) Orthopedic surgery

Q) Otolaryngology – Head and neck surgery

R) Pediatrics

S) Physical medicine and rehabilitation

T) Plastic surgery

U) Psychiatry

V) Public health and preventive medicine

W) Radiation Oncology

X) Urology

Y) Vascular Surgery

Z) Did not match this year

**2. Stressors**

1. Over the past few weeks, within the context of the COVID-19 pandemic, your stress level was:
2. Higher than usual
3. Similar to usual
4. Less than usual
5. Has the current pandemic led you to reconsider your choice of residency?

A) Yes

B) No

1. If you answered "yes" to the previous question, which of the following statements best describes your situation?
2. I have a greater interest in microbiology.
3. I have a greater interest in public health.
4. I have a greater interest in emergency medicine.
5. I have a greater interest in intensive care medicine.
6. I have a greater interest in a speciality with fewer risk of contagion.
7. None of the previous statements apply to me.
8. Has the current pandemic led you to question your choice of medicine as a career?
9. Yes
10. No
11. On a scale of 0 to 5, 0 being not stressful and 5 being an extreme level of stress, how did the following factors impact you over the past few weeks within the context of the COVID-19 pandemic?

| Potential sources of stress during the COVID-19 pandemic | **No stress** |  |  |  | **Extreme stress** | **Not Applicable** |
| --- | --- | --- | --- | --- | --- | --- |
|  | **1** | **2** | **3** | **4** | **5** |  |
| Fear of contracting COVID-19 or of contaminating someone else |  |  |  |  |  |  |
| Suspension of clerkship rotations |  |  |  |  |  |  |
| Uncertainty concerning the date of return to rotations |  |  |  |  |  |  |
| Modalities of return to rotations (e.g., lack of supervision, lack of learning opportunities) |  |  |  |  |  |  |
| CaRMS applications  (only junior clerks) |  |  |  |  |  |  |
| Feeling the need to use this time to be productive/get implicated in view of CaRMS  (only junior clerks) |  |  |  |  |  |  |
| Not having enough letters of recommendation or having letters of poorer quality  (only junior clerks) |  |  |  |  |  |  |
| CaRMS application deadline  (only junior clerks) |  |  |  |  |  |  |
| Cancellation of electives  (only junior clerk) |  |  |  |  |  |  |
| Not being able to complete rotations outside of your home university  (only junior clerk) |  |  |  |  |  |  |
| Having to take the MCCQE after the beginning of residency  (only senior clerks) |  |  |  |  |  |  |
| Lack of time to study for the MCCQE  (only senior clerks) |  |  |  |  |  |  |
| Graduating earlier to work as a resident and help with the crisis  (Only senior clerks) |  |  |  |  |  |  |
| Starting residency in the context of the pandemic and therefore potentially being exposed to severe cases of COVID-19  (only senior clerks) |  |  |  |  |  |  |

**3- WHO (five) Well-Being Index**

Please indicate for each of the five statements which is closest to how you have been feeling over the last two weeks. Notice that higher numbers mean better well-being.

| Over the last two weeks | All of the time (5) | Most of the time (4) | More than half of the time (3) | Less than half of the time (2) | Some of the time (1) | At no time (0) |
| --- | --- | --- | --- | --- | --- | --- |
| 1. I have felt cheerful and in good spirits. | 5 | 4 | 3 | 2 | 1 | 0 |
| 1. I have felt calm and relaxed. | 5 | 4 | 3 | 2 | 1 | 0 |
| 1. I have felt active and vigorous. | 5 | 4 | 3 | 2 | 1 | 0 |
| 1. I woke up feeling fresh and rested. | 5 | 4 | 3 | 2 | 1 | 0 |
| 1. My daily life has been filled with things that interest me. | 5 | 4 | 3 | 2 | 1 | 0 |

**4. Stress management and resources**

1. Have you used any university resources to overcome difficult moments during the COVID-19 pandemic; and if yes, did you find them helpful?

A) Yes, I have used university resources and I found them helpful.

B) Yes, I have used university resources, but I did not find them helpful.

C) No, I did not use any university resource.

2. If you answered “Yes” to the previous question, what university resource(s) did you use?

A)Student support network

B)Student association

C)Members of your faculty

D)Support line

E)Mobile application or other online resources

F)Other (specify) : _____________________

3) For each of the following solutions, please indicate your level of agreement:

|  | Strongly agree | Agree | Neither agree nor disagree | Disagree | Strongly disagree | Not applicable |
| --- | --- | --- | --- | --- | --- | --- |
| Returning to rotations only at the end of the pandemic |  |  |  |  |  |  |
| Returning to rotations once COVID-19 cases stop increasing |  |  |  |  |  |  |
| Catching up on missed courses with workshops, simulation activities, or other novel learning methods |  |  |  |  |  |  |
| Having training sessions on the clinical management of COVID-19 cases |  |  |  |  |  |  |
| Postponing CaRMS deadline (only junior clerks) |  |  |  |  |  |  |
| Having flexible selection criteria for CaRMS that do not penalize the lack of elective rotations  (only junior clerks) |  |  |  |  |  |  |
| Having flexible selection criteria for CaRMS allowing to submit fewer reference letters  (only junior clerks) |  |  |  |  |  |  |
| Having CaRMS interviews online (only junior clerks) |  |  |  |  |  |  |
| Having the opportunity to choose between several periods of time to take the MCCQE  (only senior clerks) |  |  |  |  |  |  |
| Having protected time during residency to study for the MCCQE  (only senior clerks) |  |  |  |  |  |  |

4. Do you have any other solutions to suggest?

_______________________________________________________________________________________________________________________________________________________________________________________________________________________
